# Structure of the human-heart fatty-acid-binding protein 3 in complex with the fluorescent probe 1-anilinonaphthalene-8-sulphonic acid

**DOI:** 10.1107/S0909049513021298

**Published:** 2013-10-01

**Authors:** Mika Hirose, Shigeru Sugiyama, Hanako Ishida, Mayumi Niiyama, Daisuke Matsuoka, Toshiaki Hara, Eiichi Mizohata, Satoshi Murakami, Tsuyoshi Inoue, Shigeru Matsuoka, Michio Murata

**Affiliations:** a JST, ERATO, Lipid Active Structure Project, 1-1 Machikaneyama-cho, Toyonaka 560-0043, Japan; b Graduate School of Science, Osaka University, 1-1 Machikaneyama-cho, Toyonaka 560-0043, Japan; c Graduate School of Engineering, Osaka University, 2-1 Yamadaoka, Suita 565-0871, Japan; d Graduate School of Bioscience and Biotechnology, Tokyo Institute of Technology, Nagatsuta, Midori-ku, Yokohama, Kanagaw 226-8501, Japan

**Keywords:** X-ray structure, FABP3–ANS complex, human-heart fatty-acid-binding protein

## Abstract

Heart-type fatty-acid-binding protein (FABP3), which is a cytosolic protein abundantly found in cardiomyocytes, plays a role in trafficking fatty acids throughout cellular compartments by reversibly binding intracellular fatty acids with relatively high affinity. The fluorescent probe 1-anilinonaphthalene-8-sulfonate (ANS) is extensively utilized for examining the interaction of ligands with fatty-acid-binding proteins. The X-ray structure of FABP3 was determined in the presence of ANS and revealed the detailed ANS-binding mechanism. Furthermore, four water molecules were clearly identified in the binding cavity. Through these water molecules, the bound ANS molecule forms indirect hydrogen-bond interactions with FABP3. The adipocyte-type fatty-acid-binding protein (FABP4) exhibits 67% sequence identity with FABP3 and its crystal structure is almost the same as that of FABP3. However, FABP4 can bind with a higher affinity to ANS than FABP3. To understand the difference in their ligand specificities, a structural comparison was performed between FABP3–ANS and FABP4–ANS complexes. The result revealed that the orientation of ANS binding to FABP3 is completely opposite to that of ANS binding to FABP4, and the substitution of valine in FABP4 to leucine in FABP3 may result in greater steric hindrance between the side-chain of Leu115 and the aniline ring of ANS.

## Abstract 2

The crystal structure of human-heart-type fatty-acid-binding protein in complex with anilinonaphthalene-8-sulfonate was solved at 2.15 Å resolution revealing the detailed binding mechanism of the fluorescent probe 1-anilinonaphthalene-8-sulfonate.

## 1. Introduction

**Figure 1:**
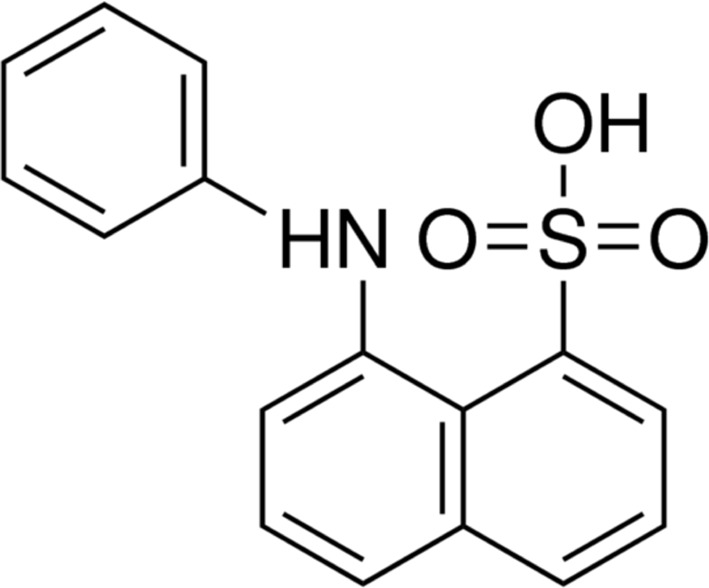
Chemical structure of 1-anilinonaphthalene-8-sulfonate (ANS).

The fluorescent probe 1-anilinonaphthalene-8-sulfonate (ANS; Fig. 1 ▶) has been known to be a competitive inhibitor for the binding of fatty acids (FAs) (Kane & Bernlohr, 1996 ▶; Kurian *et al.*, 1996 ▶). ANS is also a versatile probe to study the character of different regions of globular and membrane proteins (Slavík *et al.*, 1982 ▶). Therefore, ANS is widely used as a fluorescent probe for studying the interaction of ligands with FA-binding proteins (FABPs) (Kirk *et al.*, 1996 ▶; Zimmerman *et al.*, 2001 ▶). The dissociation constants for ANS binding to the FABPs range from 500 n*M* to 5 µ*M* (Ory & Banaszak, 1999 ▶).

FABPs belong to the intracellular lipid-binding protein (iLBP) family and are involved in reversibly binding intracellular hydrophobic ligands such as FAs. FABPs are also essential for trafficking them throughout cellular compartments, including the peroxisomes, mitochondria, endoplasmic reticulum and nucleus. To date, the main known FABPs are found in different cell types; for example, liver-type FABP (FABP1), intestinal-type FABP (FABP2), heart-type FABP (FABP3), adipocyte-type FABP (FABP4), epidermal-type FABP (FABP5), ileal-type FABP (FABP6), brain-type FABP (FABP7) and testis-type FABP (FABP9) (Offner *et al.*, 1986 ▶; Coe & Bernlohr, 1998 ▶). Each FABP has its own sequence, shows 22–73% similarity in amino acid sequence, and exhibits distinct ligand preferences, although all of the FABPs share a common structure consisting of ten antiparallel β-strands that are capped by a pair of α-helices (Sacchettini *et al.*, 1989 ▶; Scapin *et al.*, 1992 ▶; LaLonde *et al.*, 1994 ▶; Young *et al.*, 1994 ▶; Zimmerman & Veerkamp, 2002 ▶; Zhang *et al.*, 2003 ▶). Indeed, the conformation of the bound FA molecules varies among the different FABP types (Banaszak *et al.*, 1994 ▶; Thompson *et al.*, 1997 ▶; Storch & McDermott, 2009 ▶), which is thought to be due to their different metabolic roles. However, the specific function of individual FABPs has not been fully elucidated yet.

**Figure 2:**
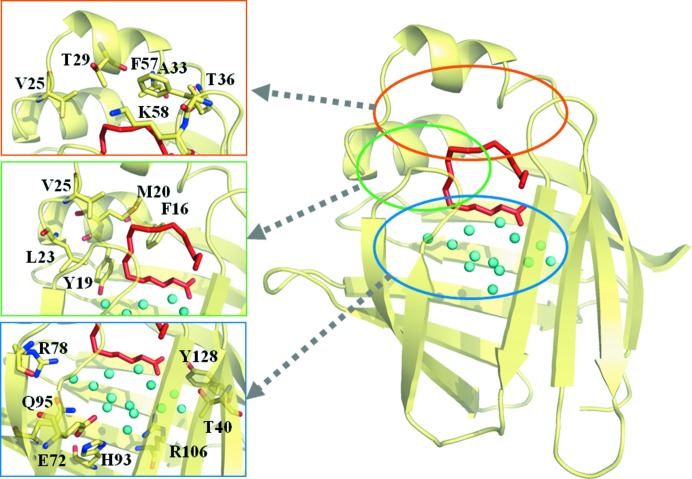
The structure of hFABP3 complexed with stearate (PDB ID code 1hmt) (Young *et al.*, 1994 ▶). ANS is shown in red. The binding cavity is divided into three sections (top, middle and bottom regions). The top region is shown in orange, the middle region in green, and the bottom region in blue.

X-ray diffraction and NMR spectroscopy have provided a great deal of interesting information about the three-dimensional structures of FABP3 (Zanotti *et al.*, 1992 ▶; Young *et al.*, 1994 ▶) and FABP4 (Xu *et al.*, 1993 ▶; Gillilan *et al.*, 2007 ▶). For instance, the bound FAs have U-shaped or L-shaped conformations in the cavity. The spacious void for FA binding in the protein interior contains many highly ordered solvent molecules that rest above a hydrophobic cluster constructed by the side-chains of aliphatic and aromatic amino acids. The binding cavity is divided into three sections: a scaffold of polar and ionizable groups that interact with the bound water molecules at the bottom, a cluster of hydrophobic side-chains in the middle part, and a mixture of these residue types at the top (Fig. 2 ▶). Furthermore, FABP3 exhibits 67% sequence identity to FABP4, and their three-dimensional structures are almost superimposable; in particular, the residues of the active sites are well conserved between the two proteins. However, FABP3 shows a lower affinity for ANS than FABP4 does (Veerkamp *et al.*, 1999 ▶). Although the three-dimensional structure of the FABP4–ANS complex has already been analyzed in a previous study (Ory & Banaszak, 1999 ▶), it is not well understood why the two proteins exhibit such different ligand specificity. Therefore, a crystallographic study of FABP3 with the bound ANS was performed to elucidate the detailed mechanism of ANS recognition and the differences in the binding specificity between FABP3 and FABP4. In this paper, we report the molecular structure of the FABP3–ANS complex determined at 2.15 Å resolution by X-ray analysis.

## 2. Materials and methods

### 2.1. Protein expression

The gene of human FABP3 (hFABP3) was synthesized with an Nde1 site at the N-terminus and a BamH1 site at the C-terminus. The 399-bp fragment was ligated into the Nde1/BamH1-digested pET15b vector (Novagen). The plasmid contains an N-terminal hexahistidine tag (His-tag) and thrombin cleavage site. The expression vector (pET15b hFABP3) was used to transform *Escherichia coli* BL21 (DE3) and the cell cultures were grown at 310 K. When the optical density of the medium reached 0.5 at 600 nm, hFABP3 expression was induced by adding 0.5 m*M* (final concentration) isopropyl β-d-1-thiogalactopyranoside to the culture medium, and the cultivation was continued at 310 K for 3 h. The cells were harvested by centrifugation and then disrupted by sonication.

### 2.2. Purification

The supernatant containing His-tagged hFABP3 was diluted four times with equilibration buffer (20 m*M* Tris-HCl, 500 m*M* NaCl, pH 7.6). The diluted supernatant was applied to a His-Trap nickel-affinity column (GE Healthcare). His-tagged hFABP3 was eluted with a linear 20 to 300 m*M* imidazole gradient in buffer (20 m*M* Tris-HCl, 500 m*M* NaCl, pH 7.6). After dialysis against buffer (20 m*M* Tris-HCl, 100 m*M* NaCl, pH 8.0), His-tagged hFABP3 was digested with thrombin (Novagen) for 22 h at 295 K to remove the N-terminal His-tag region. A further step of purification by size-exclusion column chromatography and delipidations by refolding was carried out. The fractions containing hFABP3 were pooled.

### 2.3. Crystallization of hFABP3 complexed with ANS

The hFABP3 samples in complex with ANS were prepared by the following procedure. The purified hFABP3 samples in buffer (20 m*M* phosphate, 0.05% azide, pH 7.4) at a concentration of 2 mg ml^−1^ were then incubated for 1 h at 310 K with a 1.5-fold molar ratio of ANS. Finally, the hFABP3 samples in complex with ANS were concentrated to approximately 25 mg ml^−1^ for crystallization. The crystals were obtained using the hanging-drop vapor-diffusion method. The crystals of the hFABP3–ANS complex used for X-ray diffraction were each grown in 1.0 µl of protein solution containing 10 m*M* ANS and 1 µl of reservoir solution (100 m*M* MES, 30% PEG3350, pH 6.0) equilibrated against 1.0 ml of the reservoir solution at 293 K. These crystals were directly flash-cooled in a stream of cold nitrogen gas at 100 K with buffer solution (100 m*M* MES, 43% PEG3350, pH 6.0). X-ray diffraction (XRD) data were collected on beamlines BL38B1 and BL44XU of the SPring-8 synchrotron radiation source (Harima, Japan). Diffraction data were processed using the *HKL2000* program (Otwinowski & Minor, 1997 ▶). Table 1 ▶ summarizes the data collection statistics.

### 2.4. Structure determination and refinement

The structure of the hFABP3–ANS complex was determined by the molecular replacement technique with the program *MOLREP* (Vagin & Teplyakov, 1997 ▶) using the structure of the hFABP3–stearate complex (PDB ID code 1hmt) (Young *et al.*, 1994 ▶) as a search model. The model was improved manually using the program *COOT* (Emsley & Cowtan, 2004 ▶) and was refined using the program *REFMAC* (Brünger *et al.*, 1998 ▶). The geometry of the refined model was validated by the program *PROCHECK* (Laskowski *et al.*, 1993 ▶). The structure was refined to 2.15 Å resolution. Refinement statistics are summarized in Table 2 ▶. Figures were prepared using *PyMOL* (DeLano, 2005 ▶) or *LIGPLOT* (Wallace *et al.*, 1995 ▶). The final atomic coordinates and structure-factor amplitudes (PDB entry 3wbg) have been deposited in the Worldwide Protein Data Bank (wwPDB; http://www.wwpdb.org) and the Protein Data Bank Japan at the Institute for Protein Research, Osaka University, Suita, Osaka, Japan (PDBj; http://www.pdbj.org/).

## 3. Results and discussion

### 3.1. Structure of the hFABP3–ANS complex

**Figure 3:**
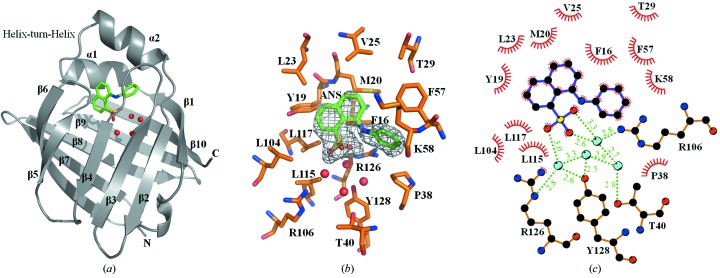
ANS interaction sites on hFABP3. (*a*) A cartoon representation of the hFABP3–ANS complex structure. ANS is represented as green sticks. The solvent molecules are represented as red spheres. (*b*) The *F*_obs_ − *F*_calc_ electron density omit map of ANS. ANS (green) is located in the binding cavity of hFABP3. The electron density is calculated at 2.15 Å and contoured at 2.5σ. The O, N and S atoms are shown in red, blue and yellow, respectively. (*c*) Principal interaction sites of hFABP3 are shown schematically. One of the ANS sulfonate O atoms forms hydrogen bonds with the hydroxyl group of Tyr128 as well as the guanidium N atom of Arg126 *via* water molecules. Furthermore, the rest of the O atoms form hydrogen bonds with the guanidium N atom of Arg106 *via* a water molecule. Hydrogen-bonding interactions are shown as green dashed lines (lengths in Å). The schematic was drawn using the program *LIGPLOT*.

The X-ray crystal structure of hFABP3 in complex with ANS was solved at 2.15 Å resolution with a crystallographic *R*_cryst_ of 22.1% (*R*_free_ = 29.1%). Our crystallographic properties and refinement statistics of the structure are presented in Tables 1 ▶ and 2 ▶. The resolution extended to 2.15 Å and was 89.3% complete with an overall *I*/σ(*I*) value above 3.0. The final structure of the hFABP3–ANS complex contains 132 of the 136 expected residues, four ANS molecules and 174 water molecules. The crystal contains four identical hFABP3 molecules in an asymmetric unit. Their root-mean-square (r.m.s.) deviations for the superimposed 132 Cα atoms are as low as 0.60 Å. There is no substantial difference among the backbone structures of the four independent molecules. We confirmed that hFABP3 was intact by MALDI MS analysis of dissolved crystals. This result demonstrates that the remaining residues are not cleaved in this region and are disordered in the crystal. The structure of hFABP3 is comprised of ten antiparallel β-barrel (β1–β10) structures and two α-helices (α1–α2) containing a ligand-binding pocket (Fig. 3*a* ▶). This binding pocket is capped by these two α-helices with an N-terminal helix–turn–helix motif which is thought to act as the regulatory portal for the binding of FA.

### 3.2. ANS binding site

Crystals of the hFABP3–ANS complex were produced by cocrystallization. The *F*_obs_ − *F*_calc_ omit map at 2.15 Å resolution clearly showed an electron density consistent with ANS (Fig. 3*b* ▶), which is located in the FA binding cavity. The same densities have been found within all four molecules in the asymmetric unit, indicating that the bound ANS molecules adopted an identical conformation. The overall positioning of ANS relative to the Cα backbone model of hFABP3 is illustrated in Fig. 3(*a*) ▶. An important feature is that ANS is located in the middle part of the binding cavity lined with a cluster of hydrophobic side-chains. In particular, the aniline and naphthalene group of ANS is surrounded by the side-chains of Phe16, Tyr19, Met20, Leu23, Val25, Thr29, Pro38, Phe57, Lys58, Leu104, Leu115 and Leu117 through hydrophobic interactions, in a manner similar to that previously observed for four FAs (Fig. 3*c* ▶) (Zanotti *et al.*, 1992 ▶; Young *et al.*, 1994 ▶). Another important feature in the binding site is that the crystal structure of the hFABP3–ANS complex clearly revealed four water molecules, which were not observed in the binding cavity of a mouse FABP4 (mFABP4) complex structure with ANS (PDB code 2ans) (Ory & Banaszak, 1999 ▶) and of the hFABP3 complex structures with C16 or C18 FAs (Zanotti *et al.*, 1992 ▶; Young *et al.*, 1994 ▶). Through these solvent molecules, the negative sulfonate group of the ANS molecule interacts with the side-chains of Thr40, Thr53, Arg106, Arg126 and Tyr128 by forming a hydrogen-bonding cluster (Fig. 3*c* ▶). However, in the FA complex, the carboxylate group of the four FAs (palmitate, oleate, stearate and elaidate) forms direct hydrogen-bond interactions with the side-chains of Arg126 and Tyr128 (Zanotti *et al.*, 1992 ▶; Young *et al.*, 1994 ▶). Indeed, the sulfonate group of ANS is positioned far away from Arg126 (6.4 Å) and Tyr128 (6.5 Å) so that their direct interaction is highly unlikely in the hFABP3–ANS complex. Therefore, to compensate for this distance, these water molecules are located at similar positions as those of the FA carboxylate group, resulting in interactions with the side-chains of the Arg and Tyr residues. These results suggest that other hydrophobic ligands with a negative group may also be recognized by hFABP3 in a similar binding manner.

### 3.3. Comparison with the mFABP4–ANS complex

**Figure 4:**
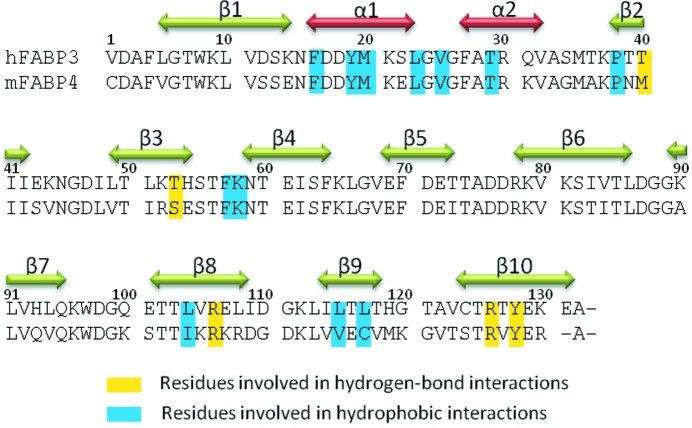
Sequence alignment of hFABP3 and mFABP4.

The sequences of hFABP3 and mFABP4 exhibit a 67% identity (Fig. 4 ▶). When the structures of hFABP3–ANS and mFABP4–ANS complexes are optimally superimposed, the r.m.s. deviation value is evaluated to be an average of 0.66 Å for the 130 Cα atoms. The value indicates that the three-dimensional structure of the hFABP3–ANS complex is most similar to that of the mFABP4–ANS complex. In particular, the active residues in the binding cavity are well conserved between hFABP3 and mFABP4; ten of the 16 relevant residues of hFABP3 shown in Fig. 3(*c*) ▶, Phe16, Tyr19, Val25, Thr29, Pro38, Phe57, Lys58, Arg106, Arg126 and Tyr128, are conserved in mFABP4.

**Figure 5:**
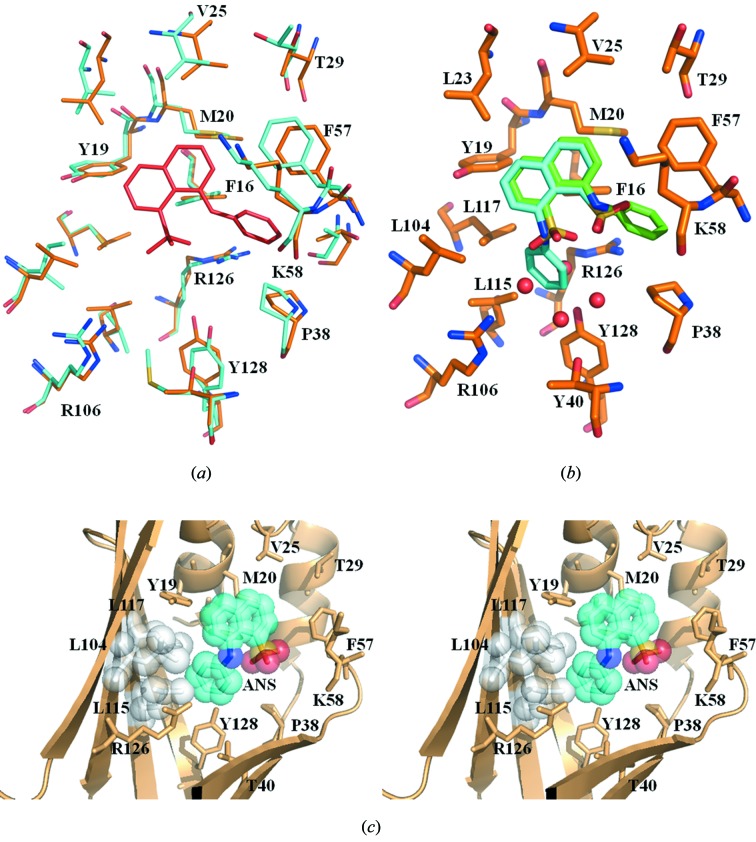
Superimposition of mFABP4 on hFABP3 with ANS. These views were obtained by superimposing the main-chain atoms of hFABP3 and mFABP4. (*a*) This view is drawn without ANS binding to mFABP4. mFABP4, hFABP3 and ANS are shown in blue, yellow and red, respectively. Only the number of the corresponding residues between hFABP3 and mFABP4 are shown. (*b*) This view is drawn without the active residues of mFABP4. hFABP3, ANS in hFABP3, and ANS in mFABP4 are shown in yellow, green and blue, respectively. (*c*) This stereoview is drawn without ANS binding to hFABP3 and active residues of mFABP4. L104, L115 and L117 of hFABP3 and ANS in mFABP4 are shown in white and blue spheres, respectively.

Our superimposition of mFABP4 to hFABP3 with ANS also indicates that the conserved active residues in mFABP4 are located in almost the same place as those in hFABP3, as shown in Fig. 5(*a*) ▶. This suggests that ANS may bind to mFABP4 in almost the same manner as to hFABP3. However, our result clearly demonstrated that the orientation of ANS binding to hFABP3 is exactly the opposite of that of ANS binding to mFABP4 (Fig. 5*b*) ▶. The present structure, highly refined at 2.15 Å resolution, tempted us to examine how the two proteins could exhibit such different ligand specificities.

In the three-dimensional structure of the mFABP4–ANS complex, the aniline group of ANS is well surrounded by the side-chains of Pro38, Met40, Ser53, Ile104, Val115, Cys117, Arg126 and Tyr128. The amino acid sequences of hFABP3 and mFABP4 were compared and aligned (Fig. 4 ▶). Three residues (Pro38, Arg126 and Tyr128) of them are invariant in hFABP3. Since the substitutions at two positions (Met40 by Thr40 and Ser53 by Thr53) do not disturb ANS binding, they seem to have little effect on ANS binding (Fig. 5*b* ▶). Therefore, the ligand specificity of hFABP3 may be caused mainly by the substitutions at the remaining three residues (Ile104 by Leu104, Val115 by Leu115, Cys117 by Leu117). In particular, Val115 in mFABP4, which forms the hydrophobic interaction with the aniline group of ANS, is replaced with Leu115 in hFABP3. This substitution may be one of the major factors for the difference in orientation of ANS binding to hFABP3 and mFABP4, because the distance between the side-chain of Leu115 and the aniline ring of ANS is 2.2 Å, which generates a large steric hindrance (Fig. 5*c* ▶).

## 4. Conclusion

We solved the crystal structure of hFABP3 in complex with ANS at 2.15 Å resolution. The structure clarified four water molecules in the binding cavity of hFABP3, and through these water molecules the bound ANS molecule forms a hydrogen-bonding network with the side-chains of Thr40, Thr53, Arg106, Arg126 and Tyr128. In addition to van der Waals interactions, these indirect interactions should contribute to ANS recognition. Furthermore, our analysis revealed that the orientation of ANS binding to hFABP3 is opposite to that of ANS binding to mFABP4. The structural comparison of hFABP3 and mFABP4 suggested that one of the causes is the effect of steric hindrance between the side-chain of Leu115 and the aniline ring of ANS, arising from the replacement of Val115 of mFABP4 by Leu115 of hFABP3.

## Supplementary Material

PDB reference: 3wbg

## Figures and Tables

**Table 1:** Data collection statistics for the FABP3–ANS complex Values given in parentheses are for the highest-resolution shell.

Space group	*C*2
Unit-cell parameters (Å, °)	*a* = 127.1, *b* = 29.3, *c* = 140.7, α = 90, β = 112.4, γ = 90
Resolution range (Å)	50–2.15 (2.19–2.15)
No. of molecules per asymmetric unit	4
*V*_M_ (Å^3^ Da^−1^)	1.7
Solvent content (%)	31.0
No. of reflections	87096
No. of unique reflections	24494
Average redundancy	3.6 (3.7)
*I*/σ(*I*)	7.7 (3.1)
*R*_merge_ (%)†	6.3 (33.0)
Completeness (%)	90.0 (89.3)

† *R*_merge_ = ∑_*hkl*_∑_*i*_|*I*_*i*_(*hkl*) − 〈*I*(*hkl*)〉|/∑_*hkl*_∑_*i*_ *I*_*i*_(*hkl*), where *I*_*i*_(*hkl*) is the *i*th observed intensity of reflection *hkl* and 〈*I*(*hkl*)〉 is the average intensity over symmetry-equivalent measurements.

**Table 2:** Refinement statistics

Resolution range (Å)	29.85–2.15
No. of reflections	23179
No. of protein atoms (non-hydrogen)	4169
No. of ligands	4
No. of water molecules	174
*R*_cryst_ (%)†	22.1
*R*_free_ (%)‡	29.1
R.m.s.§ deviations in bond length (Å)	0.01
R.m.s.§ deviations in bond angles (°)	1.82

† *R*_cryst_ = ∑||*F*_o_| − |*F*_c_||/∑|*F*_o_| calculated from 95% of the data, which were used during refinement.

‡ *R*_free_ = ∑||*F*_o_| − |*F*_c_||/∑|*F*_o_| calculated from 5% of the data, which were used during refinement.

§ The abbreviation used is root mean square (r.m.s.).
